# First study with positive cardiovascular outcome in obesity: Reflections on SELECT

**DOI:** 10.1002/agm2.12300

**Published:** 2024-05-18

**Authors:** Qi Pan, Sijia Fei, Ting Xie, Lixin Guo

**Affiliations:** ^1^ Department of Endocrinology, Beijing Hospital, National Center of Gerontology, Institute of Geriatric Medicine Chinese Academy of Medical Sciences Beijing China

## 1 BACKGROUND

### 1.1 Clinical dilemmas: there is a heavy burden of cardiovascular diseases (CVD) in obesity, and medications for weight management while reducing cardiovascular risks are not yet clinically available

Obesity, a recurring and progressive chronic disease, has emerged as a significant global public health concern, imposing substantial burdens on both human health and socioeconomic development. Recent data predict a sharp rise in the global overweight or obesity rate, from 38% in 2020 to an anticipated 51% by 2035, positioning half of the global population in these categories.^1^ At present, about 50% of adults in China are overweight or obese, marking it as the country with the highest number of such cases worldwide. The prevalence peaks in the age groups of 55–59 and 70–74. The risk of cardiometabolic complications (non‐alcoholic fatty liver disease, prediabetes, dyslipidemia, and hypertension, etc.) is significantly higher in overweight and obese individuals compared to those with normal body mass index (BMI).^2^ In addition, overweight and obesity are independent risk factors for cardiovascular risks.^3^ According to the 2015 Global Burden of Disease (GBD) data, an estimated 2/3 of cardiovascular deaths are attributed to high BMI,^4^ and the risk of coronary heart disease and stroke increases by 27% and 18% respectively, for every 5 kg/m^2^ increase in BMI.^5^

Currently, guidelines and consensus primarily recommend three treatments for managing overweight and obesity: lifestyle interventions, pharmacotherapy, and bariatric surgery.^6^, ^7^, ^8^ Lifestyle interventions are the initial approach, yet challenges such as low adherence, modest weight loss, and rebound gain persist. Additionally, there is a lack of universally recognized lifestyle intervention programs in China.^9^ Studies confirmed that intensive lifestyle interventions focused primarily on weight loss did not reduce the risk of cardiovascular events.^10^ Bariatric surgery can greatly reduce weight and improve cardiometabolic health in a short period. However, it also presents challenges such as poor acceptance, postoperative complications, and an incomplete industry standard and policy system.^11^ For those not responding to lifestyle changes, weight‐loss medications are suggested. Despite the global approval and marketing of various drugs, concerns over cardiovascular risks, carcinogenicity, and mental health effects have led to market withdrawals of some, including sibutramine, which was found to significantly increase the risk of myocardial infarction and stroke.^12^ Consequently, in 2012, the Food and Drug Administration (FDA) promulgated the “Cardiovascular Risk Assessment Requirements for Weight‐Loss Drugs,” making the Cardiovascular Outcomes Trials (CVOT) a mandatory study for the approval of weight‐loss medications. To date, no weight management medication approved globally has been confirmed by CVOT to reduce body weight while improving cardiovascular outcomes.

The SELECT (semaglutide and cardiovascular outcomes in obesity without diabetes)—an international multicenter CVOT of once‐weekly semaglutide 2.4 mg—potentially provides a solution to this clinical challenge. The results^13^ were reported at the American College of Cardiology (ACC) Annual Meeting in November 2023 and published simultaneously in the *New England Journal of Medicine*.

### 1.2 Study design and main results

The SELECT trial is an international, multicenter (41 countries and areas), randomized, double‐blind, parallel, placebo‐controlled, Phase III clinical trial. It is aimed at evaluating the effectiveness of semaglutide 2.4 mg versus placebo in reducing major adverse cardiovascular events (MACE) among patients with established CVD and overweight or obese and without T2DM, all receiving the standard of care.

A total of 17 604 patients with overweight or obesity were enrolled. The mean age of patients was 61.6 ± 8.9 years, 12 732 patients (72.3%) were male, and 6698 (38.0%) were elderly patients aged ≥65 years. The average BMI was 33.3 ± 5.0 kg/m^2^, with 71.5% being classified as obese (BMI ≥30 kg/m^2^). The average follow‐up duration was 39.8 months.

Regarding efficacy endpoints: compared with placebo, semaglutide 2.4 mg significantly reduced the risk of MACE (cardiovascular death, and non‐fatal myocardial infarction and non‐fatal stroke) by 20% (HR, 0.80; 95% CI 0.72–0.90; *p* < 0.001). Moreover, the pre‐specified baseline heterogeneity analysis showed consistency in the trend of MACE benefits in the two age subgroups of 65–74 and ≥75 years.

On the safety front: the rate of gastrointestinal events leading to discontinuation in the semaglutide group was higher compared to the placebo (10.0% vs. 2.0%, *p* < 0.01). Nevertheless, the semaglutide group had a lower incidence of serious adverse events (33.4% vs. 36.4%, *p* < 0.001). Importantly, the study found no increase in serious adverse events related to gastrointestinal diseases, acute kidney failure, pancreatitis, tumors, or mental health disorders in patients treated with semaglutide compared to those receiving the placebo.

## 2 STUDY CHARACTERISTICS AND FINDINGS

### 2.1 Study characteristics

SELECT is the first CVOT to confirm the cardiovascular benefit of weight management drugs globally. Unlike previous cardiovascular outcome studies of GLP‐1RAs,^14^ the population included in this study were non‐T2DM patients, thus excluding the effect of T2DM on CVOT results. Compared with the SUSTAIN6 (Semaglutide and Cardiovascular Outcomes in Patients with T2DM), a CVOT investigating the efficacy of semaglutide 1.0 mg in patients with T2DM and at high cardiovascular risk,^15^ more patients with CVD and overweight or obesity were included in the SELECT study (17 604 vs. 3297), and they received a higher dose of semaglutide (2.4 mg) with a longer follow‐up (39.8 months vs. 25.2 months) duration.

### 2.2 Notable findings and reflections

STEP (semaglutide 2.4 mg once a week in adults with overweight or obesity) Programme ^16^ confirmed that semaglutide 2.4 mg effectively reduced the body weight by more than 10% compared to baseline, and a maximum reduction of 17.4% in patients with overweight or obesity. The results from the SELECT study suggested the cardiovascular benefit of semaglutide 2.4 mg in patients with established CVD and overweight or obesity.^13^ However, is this benefit solely attributed to the weight loss?

A prior study indicated that for individuals who were overweight or obese, a minimum weight reduction of 10% is essential to diminish cardiovascular risks.^17^ However, in the SELECT study, patients administered semaglutide 2.4 mg experienced an average weight loss of 9.4%, falling short of the 10% threshold. This level of weight loss was different from that achieved in the STEP Programme, which may be related to the design of the primary endpoints of the two studies. Since the primary endpoint of SELECT was the time to the first major adverse cardiovascular event, rather than the level of weight loss. Therefore, the study allowed for dose adjustments based on patient tolerance and compliance, resulting in approximately 23% of patients not reaching the 2.4 mg dosage at the end of the trial. Moreover, the early manifestation of the between‐group difference in MACE suggests that the cardiovascular benefits of semaglutide 2.4 mg may stem from the prompt improvement in cardiometabolic factors (such as blood pressure, blood glucose, lipid profile, and inflammation) associated with cardiovascular outcomes, rather than solely from the weight loss. Non‐clinical studies have shown that GLP‐1RA can impact the development and progression of atherosclerosis in animals with and without diabetes, by improving endothelial function, reducing inflammation, decreasing the migration and deformation of vascular smooth muscle cells, promoting plaque stability, and lessening platelet aggregation.^18^ Additionally, semaglutide may also achieve cardiovascular protection by reducing epicardial and perivascular adipose tissues with proinflammatory and proatherogenic effects.^19^, ^20^

In the SELECT study, the baseline utilization rates for lipid‐lowering medications, antiplatelet agents, and beta blockers stood at 90.1%, 86.5%, and 70.2%, respectively. Beyond the standard care, semaglutide 2.4 mg additionally lowered the average systolic blood pressure, waist circumference, low‐density lipoprotein (LDL) cholesterol levels, and high‐sensitivity C‐reactive protein (hsCRP) levels by 3.82 mmHg, 7.56 cm, 5.25%, and 39.1%, respectively, culminating in a 20% reduction in major adverse cardiovascular events (MACE). Therefore, it is suggested that semaglutide may reduce the residual risk of CVD to some extent, especially residual inflammatory risk. Previous research has indicated that within the context of obesity, an increase in visceral adipose tissue leads to a decrease in adiponectin levels, a rise in plasma‐free fatty acid levels, and the production of numerous proinflammatory cytokines. This chronic inflammation could damage vascular endothelium, induce hemodynamic changes, and cause myocardial remodeling, thereby accelerating the development of atherosclerosis and impacting the heart's structure and function.^21^ In previous studies involving patients with overweight or obesity, such as STEP‐HFpEF (Semaglutide in Patients with Heart Failure with Preserved Ejection Fraction and Obesity)^22^ and the STEP Programme, semaglutide 2.4 mg has been shown to reduce hsCRP levels by more than 50% compared to baseline.^23^, ^24^

As previously mentioned, T2DM patients were excluded from the study, the mean HbA1c level at baseline was 5.8 ± 0.3%, and 11 696 patients (66.4%) were prediabetes (defined as a mean HbA1c level of 5.7%–6.4%), with 66.8% and 66.1% in the semaglutide and placebo groups, respectively. By the end of 104 weeks of follow‐up, the proportion of patients with HbA1c ≥6.5% in the semaglutide 2.4 mg group was lower than that in the placebo group (3.5% vs. 12.0%), which provided evidence for the primary prevention of diabetes in prediabetes patients with overweight or obesity.

Consistent with the results of previous GLP‐1RA studies,^15^, ^25^ semaglutide 2.4 mg also showed a higher incidence of gastrointestinal adverse events in SELECT, mainly including nausea, vomiting, and diarrhea. Gastrointestinal adverse events are known to be common with GLP‐1RA medications and typically occur during the initiation and dose escalation phases. Most patients can tolerate these events, but caution is needed in patients with a history of severe gastroparesis or intestinal paralysis. For special populations, especially the elderly, previous studies reported that the safety and efficacy of GLP‐1RA in the elderly population (≥65 years) were similar to that in adults. Given the gastrointestinal adverse events, caution should be exercised when administering semaglutide to elderly patients to prevent or avoid exacerbating malnutrition, sarcopenia, and frailty.^26^

## 3 CLINICAL SIGNIFICANCE OF SELECT

At present, medications of the GLP‐1RA class, such as liraglutide 3.0 mg,^27^ semaglutide 2.4 mg,^28^ and tirzepatide (GLP‐1/GIP [glucose‐dependent insulinotropic polypeptide] agonist)^29^ have been approved for weight management for adult patients with obesity or overweight with obesity‐related complications. The previous study of semaglutide 2.4 mg in obese patients with heart failure, the STEP‐HFpEF study,^22^ confirmed its significant improvement in clinical functional endpoints such as KCCQ‐CSS score (Kansas City Cardiomyopathy Questionnaire clinical summary score) and 6MWD (6‐Minute Walk Distance) for patients with heart failure and preserved/mildly reduced ejection fraction (HFpEF/HFmrEF), demonstrating the cardiovascular benefit of semaglutide 2.4 mg in patients with obesity. SELECT further confirmed that semaglutide 2.4 mg significantly reduced the risk of major cardiovascular events. As the first weight management drug with cardiovascular benefit confirmed by rigorous RCT globally, semaglutide 2.4 mg is of landmark significance and provides strong clinical evidence for the treatment of patients with CVD and overweight or obesity.

GLP‐1RAs with proven cardiovascular benefits have been recommended by the guidelines as a first‐line treatment for cardiovascular risk reduction in patients with T2DM and CVD or at high cardiovascular risk.^30^ The SELECT further provided evidence for the use of high‐dose GLP‐1RA in a broader population of patients with overweight or obesity and CVD without T2DM.

## 4 CONCLUSION

CVD remains the principal cause of mortality both in China and globally. Overweight and obesity are not only significant risk factors for CVD but also as an independent chronic disease. With societal economic progress and lifestyle alterations, overweight and obesity have increasingly emerged as major societal concerns. Effective weight management necessitates a multidisciplinary, multilevel, and long‐term comprehensive strategy that incorporates political, economic, environmental, social, and personal factors. Presently, the availability of new management options for obesity and CVD is expanding, and it is expected that these evidence‐based treatments will overcome current clinical challenges, thereby better fulfilling the demands of clinical care.

## AUTHOR CONTRIBUTIONS

QP made substantial contributions to the design of the study and drafted the manuscript. SF and TX calibrated the format and language of the article, while LG critically reviewed the manuscript. All authors have read and approved the final version to be published.

## FUNDING INFORMATION

The study was supported by National High Level Hospital Clinical Research Funding (BJ‐2022‐195).

## CONFLICT OF INTEREST STATEMENT

The authors declare that they have no competing interests.
